# Longitudinal Profiling in Phenotypic Metric of Aging: Insights From the Baltimore Longitudinal Study of Aging

**DOI:** 10.1093/geroni/igab046.017

**Published:** 2021-12-17

**Authors:** Pei-Lun Kuo, Morgan Levine, Jennifer Schrack, Michelle Shardell, Luigi Ferrucci

**Affiliations:** 1 National Institute on Aging, National Institute on Aging, Maryland, United States; 2 Yale University, New Haven, Connecticut, United States; 3 Johns Hopkins Bloomberg School of Public Health, Baltimore, Maryland, United States; 4 University of Maryland School of Medicine, Baltimore, Maryland, United States; 5 National Institute on Aging, Baltimore, Maryland, United States

## Abstract

It remains challenging to quantify the pace of aging across lifespan due to lack of comprehensive longitudinal measurements across wide range of age. In Baltimore Longitudinal Study of Aging, we have measured the longitudinal trajectories of more than 30 phenotypes across four pre-identified domain - body composition, energy regulation, homeostatic mechanisms and neurodegeneration/neuroplasticity, among participants with age between 20+ and 90+. We implemented a two-stage approach to summarize the longitudinal trajectories of these phenotypes across four domains into a summarized score. We demonstrated that higher summarized score (denoting for slower longitudinal phenotypic decline) is associated with slower decline in both cognitive and physical functions, across different stages of adulthood. Our results imply that deep longitudinal profiling contains rich information and may potentially replace diseases as an early endpoint in trials targeting at aging. Further, understanding the underpinning of longitudinal phenotypic trajectories may provide clues to the biological mechanisms of aging.

